# The association of heat shock protein genetic polymorphisms with age-related hearing impairment in Taiwan

**DOI:** 10.1186/s40463-021-00512-2

**Published:** 2021-04-29

**Authors:** Ning-Chia Chang, Hua-Ling Yang, Chia-Yen Dai, Wen-Yi Lin, Meng-Hsuen Hsieh, Chen-Yu Chien, Kuen-Yao Ho

**Affiliations:** 1 Department of Otorhinolaryngology, School of Medicine, College of Medicine, Kaohsiung Medical University, Kaohsiung, Taiwan; 2 Department of Otorhinolaryngology, Kaohsiung Municipal Siaogang Hospital, Kaohsiung, Taiwan; 3 Health Management Center, Kaohsiung Municipal Siaogang Hospital, Kaohsiung, Taiwan; 4 Department of Internal Medicine, Division of Hepatobiliary and Pancreatic Medicine, Kaohsiung Medical University Hospital, Kaohsiung, Taiwan; 5 Department of Internal Medicine, School of Medicine, College of Medicine, Kaohsiung Medical University, Kaohsiung, Taiwan; 6 Health Management Center, Kaohsiung Medical University Hospital, Kaohsiung, Taiwan; 7 Department of Otorhinolaryngology, Kaohsiung Medical University Hospital, No. 100, Tzyou 1st Road, Kaohsiung, 807 Taiwan

**Keywords:** Age-related hearing impairment, Heat shock protein, Single nucleotide polymorphism

## Abstract

**Background:**

Age-related hearing impairment (ARHI) is a major disability among the elderly population. Heat shock proteins (HSPs) were found to be associated with ARHI in animal studies. The aim of this study was to analyze the associations of single nucleotide polymorphisms (SNPs) of HSP genes with ARHI in an elderly population in Taiwan.

**Methods:**

Participants ≥65 years of age were recruited for audiometric tests and genetic analyses. The pure tone average (PTA) of the better hearing ear was calculated for ARHI evaluation. The associations of HSPA1L (rs2075800 and rs2227956), HSPA1A (rs1043618) and HSPA1B (rs2763979) with ARHI were analyzed in 146 ARHI-susceptible (cases) and 146 ARHI-resistant (controls) participants.

**Results:**

The “T” allele of HSPA1B rs2763979 showed a decreased risk of ARHI. The “TT” genotype of rs2763979 also showed a decreased risk of ARHI in the dominant hereditary model. For HSPA1L (rs2075800 and rs2227956) and HSPA1A (rs1043618), the haplotype “CAG” was related to a decreased risk of ARHI.

**Conclusion:**

These findings suggest that HSP70 polymorphisms are associated with susceptibility to ARHI in the elderly population.

**Graphical abstract:**

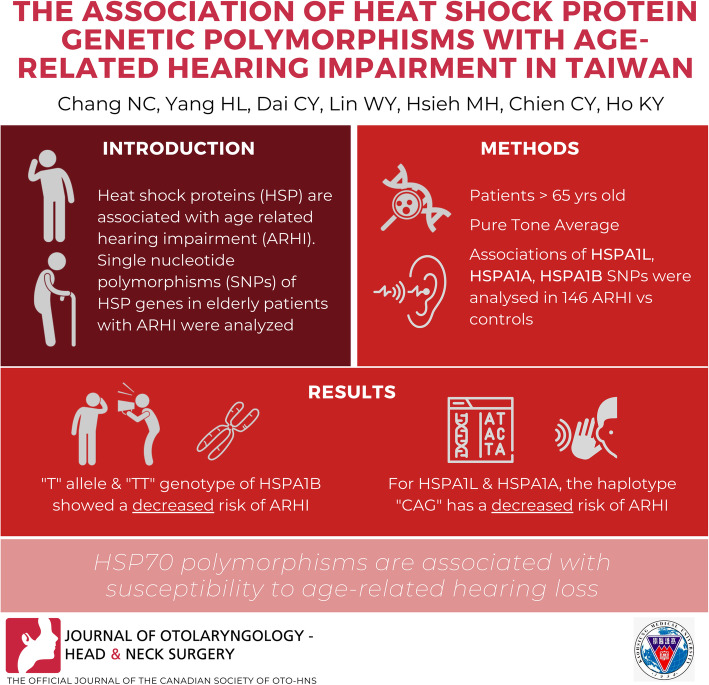

## Introduction

Age-related hearing impairment (ARHI), or presbycusis, is a common disability in the elderly population worldwide. The prevalence of hearing impairment has been reported to be 34% in people 65 years of age or older, and it increases to 72% in people 85 years of age or older [1, 2]. In Taiwan, the prevalence of hearing impairment is reportedly as high as 78% among the population 65 years of age or older [3]. The detailed mechanisms of ARHI development remain unclear. It is a multifactorial condition, representing the end result of multiple intrinsic (e.g., genetic predisposition) and extrinsic (e.g., noise exposure) factors acting on the inner ear leading to the accumulation of damage in the pathway of auditory signal transduction [4]. Chronic inflammation, toxic substances, hypoxia, and noise may increase inner ear oxidative stress, increase the production of reactive oxygen species (ROS), lead to necrosis and apoptosis of inner ear cells, and result in presbycusis [4–6].

In the mechanism of presbycusis development, ROS are the major substances responsible for DNA and protein damage leading to apoptosis [4], whereas heat shock proteins (HSPs) play protective roles in preventing cell death. The 70-kDa heat shock proteins (HSP70s) are the most widely investigated HSPs in humans. The human HSP70 gene family consists of HSP70–1 (HSPA1A), HSP70–2 (HSPA1B), and HSP70-Hom (HSPA1L). The expression of both HSPA1A and HSPA1B is heat-inducible, and these two genes encode an identical protein product of 641 amino acids. HSPA1L is not heat-inducible and encodes a protein that is highly related to HSPA1A [7].

A number of studies on the relationship between HSP and noise-induced hearing loss (NIHL) have been reported, but the results are diverse and have ethnic differences [8–12]. Two meta-analyses reviewed five HSP70 polymorphisms (rs1043618, rs1061581, rs2075800, rs2227956, and rs2763979) and found that the rs1061581 and rs2227956 polymorphisms were significantly associated with NIHL in Caucasian males, but no significant association of any of the five SNPs with NIHL was found in Asian populations [13, 14]. Although there have been many studies investigating the associations of HSPs with NIHL, a limited number of studies have discussed the relationships between HSPs and ARHI. An animal study reported that the heat shock protein 1 (Hspb1) gene was downregulated in middle-aged mice with mild presbycusis, whereas it was upregulated in those with severe presbycusis [15]. Another animal study found that pharmacological upregulation of HSPs might attenuate ARHI. Dietary supplementation with geranylgeranylacetone (GGA), a heat shock protein inducer, might increase the expression of HSP in the cochlea of mice and suppress ARHI [16]. Unfortunately, no article discussing the association of HSPs with human presbycusis could be retrieved from the PubMed database. The role of HSPs in human ARHI remains unclear.

In the mechanism of ARHI proposed by Yamasoba et al. [4] and the pathway of NIHL proposed by Henderson et al. [5], these two hearing impairments shared some common pathways in the pathogenesis. Studies have reported that the genetic polymorphisms of HSP70 are associated with susceptibility to NIHL [8–14], and we proposed that the genetic polymorphisms of HSP70 might also relate to susceptibility to ARHI development. Therefore, we initiated the present study to investigate the association of HSP70 genetic polymorphisms with ARHI in an elderly population in Taiwan.

## Materials and methods

### Subjects

The participants of this study were recruited from clients who received national annual health examinations performed by the Health Management Center at a regional teaching hospital. The national annual health examinations were free of charge for subjects older than 65 years of age, with funding provided by the Health Promotion Administration, Ministry of Health and Welfare, Taiwan. We recruited volunteers who were 65 years of age or older and who agreed to participate in the study to receive additional pure tone audiometric tests and take self-reported questionnaires about their history of noise exposure and their medical history of ear diseases. Participants with dementia and those who could not sit independently to receive the audiometric tests were excluded from this study.

### Audiometric assessments

Pure-tone audiometry was performed in sound-attenuating booths by trained medical technicians using standard procedures that met the requirements of the Council of Labor Affairs, Executive Yuan, Taiwan. The audiometric data were recorded at frequencies of 500, 1000, 2000, 3000, 4000, 6000, and 8000 Hz. The speech frequency (500, 1000, 2000, and 4000 Hz) pure tone average (PTA) was calculated on each side. The PTA of the better hearing ear was used to evaluate the severity of hearing impairment in accordance with the World Health Organization’s definition of hearing loss. The participants were then divided into three groups according to the PTA levels. Participants in the worst quartile of hearing level were classified into the age-related hearing impairment group (ARHI-susceptible group), while those in the best quartile of hearing level were classified into the ARHI-resistant group. The others were classified into the intermediate group. The ARHI-susceptible and ARHI-resistant groups were chosen for case-control analysis.

### Genotyping

HSPA1A, HSPA1L and HSPA1B genetic polymorphisms rs1043618, rs2227956, rs2075800 and rs2763979 were selected as the target SNPs, which were referenced from earlier studies [10, 12–14]. Blood samples were obtained from all the participants with written informed consent. Each specimen was collected in an ethylenediaminetetraacetic acid (EDTA) tube and centrifuged (2000 *g*, 20 min). The buffy coat was isolated, and DNA was extracted using a commercial DNA extraction kit (Gentra Corp., Minneapolis, Minn, USA). Genotypes for the selected polymorphisms were screened with ABI TaqMan SNP genotyping assays (Applied Biosystems, Foster City, Calif., USA). The extracted DNA and genotyping assays were added to TaqMan universal PCR master mix (Roche, Branchburg, N.J., USA) according to the manufacturer’s instructions. The genotyping procedures were then performed by using the ABI PRISM 7500 real-time PCR system (Applied Biosystems). The results were analyzed using ABI 7500 System sequence detection software version 2.3 (Applied Biosystems).

### Statistical analysis

All data were input to a computer and analyzed using the IBM® SPSS® statistics software package version 25 (International Business Machines Corp., Armonk, N.Y., USA). Continuous data were analyzed using independent-sample Student’s t-tests. Categorical data were computed using the two-sided χ2 test. The calculation of odds ratios (ORs) and 95% confidence intervals (95% CIs) and adjustments for potentially confounding factors were performed with logistic regressions. Haplotype analysis was performed by Haploview software (https://www.broadinstitute.org/haploview/downloads) and PLINK software package version 1.07 (http://pngu.mgh.harvard.edu/purcell/plink/) [17]. The level of statistical significance was set at *p* < 0.05.

## Results

A total of 821 participants, including 464 (56.5%) men and 357 (43.5%) women, were included in the present study. The average age of the participants was 71.94 ± 5.81 years (range 65–97 years). There was no significant age difference between the sexes of the participants (t-test; *p* = 0.075). The average better hearing ear PTA was 39.96 ± 16.84 dBHL, and the average hearing was better in females than in males (t-test; *p* < 0.001). In total, 272 (33.3%) participants claimed to have occupational noise exposure before their retirement. In the participants who were exposed to occupational noise, 209 were men (45.3% of the male participants; 76.8% of the total noise-exposed participants), and 63 were women (17.7% of the female participants; 23.2% of the total noise-exposed participants). Other demographic data, including alleles and genotypes of the target SNPs, are presented in Table 1.

**Table 1:** Demographics of the participants

	Male	Female	Total
Number	464 (56.5%)	357 (43.5%)	821 (100%)
Age (years)	72.26 ± 6.00	71.52 ± 5.66	71.94 ± 5.81
Better hearing ear PTA (dBHL)	42.63 ± 16.69	36.47 ± 16.41	39.96 ± 16.84
Occupational noise exposure	209 (45.3%)	63 (17.7%)	272 (33.3%)
ARHI grouping			
ARHI susceptible	149 (32.1%)	70 (19.6%)	219 (26.7%)
Intermediate	213 (45.9%)	160 (44.8%)	373 (45.4%)
ARHI resistant	102 (22.0%)	127 (35.6%)	229 (27.9%)
Genotype			
rs1043618			
GG	229 (49.4%)	180 (50.4%)	409 (49.8%)
CG	188 (40.5%)	139 (38.9%)	327 (39.8%)
CC	35 (7.5%)	32 (9.0%)	67 (8.2%)
Undetermined	12 (2.6%)	6 (1.7%)	18 (2.2%)
rs2227956			
AA	273 (58.8%)	226 (63.3%)	499 (60.8%)
AG	161 (34.7%)	108 (30.3%)	269 (32.8%)
GG	19 (4.1%)	18 (5.0%)	37 (4.5%)
Undetermined	11 (2.4%)	5 (1.4%)	16 (1.9%)
rs2075800			
CC	174 (37.5%)	133 (37.3%)	307 (37.4%)
CT	220 (47.4%)	168 (47.1%)	388 (47.3%)
TT	66 (14.2%)	54 (15.1%)	120 (14.6%)
Undetermined	4 (0.9%)	2 (0.6%)	6 (0.7%)
rs2763979			
CC	238 (51.3%)	198 (55.5%)	436 (53.1%)
CT	185 (39.9%)	120 (33.6%)	305 (37.1%)
TT	31 (6.7%)	32 (9.0%)	63 (7.7%)
Undetermined	10 (2.2%)	7 (2.0%)	17 (2.1%)
Allele			
rs1043618			
G	646 (71.5%)	499 (71.1%)	1145 (71.3%)
C	258 (28.5%)	203 (28.9%)	461 (28.7%)
rs2227956			
A	707 (78.0%)	560 (79.5%)	1267 (78.7%)
G	199 (22.0%)	144 (20.5%)	343 (21.3%)
rs2075800			
C	568 (61.7%)	434 (61.1%)	1002 (61.5%)
T	352 (38.3%)	276 (38.9%)	628 (38.5%)
rs2763979			
C	661 (70.7%)	516 (73.7%)	1177 (73.2%)
T	274 (29.3%)	184 (26.3%)	431 (26.8%)

### Case-control study

There were 219 (26.7%) participants classified into the ARHI-susceptible group (mean age = 74.98 ± 6.51 years; average PTA = 62.04 ± 13.76 dBHL) and 229 (27.9%) participants with the best hearing, classified into the ARHI-resistant group (mean age = 69.83 ± 4.69 years; average PTA = 22.65 ± 4.40 dBHL). The average age was significantly younger in the ARHI-resistant group (t-test; *p* < 0.001). To minimize the interference of age in the analysis, we used age-matched selection to choose participants from the ARHI-susceptible group as cases and to select participants from the ARHI-resistant group as controls. In each group, 146 participants were matched. The average PTA for the cases was 60.40 ± 12.82 dBHL, while it was 22.91 ± 4.27 dBHL for the controls. The average ages for the cases and controls were 71.71 ± 4.70 years and 71.36 ± 5.00 years, respectively, without a significant difference (t-test; *p* = 0.539). The sex structure in each group was male/female = 68.5%/31.5% in the case group and 47.3%/52.7% in the control group. Females generally had better hearing than males (chi-square; *p* < 0.001). The distribution of noise exposure history (Y/N) was also different between these two groups. There were more participants with noise exposure history in the case group (42.5%) than in the control group (28.3%) (chi-square; *p* = 0.011).

### Genetic analysis

#### Allele analysis

Participants with the “T” allele of rs2763979 had a decreased risk of ARHI, regardless of the adjustments for sex and noise exposure. However, there was no significant association of the alleles of the rs1043618, rs2227956 or rs2075800 SNP with ARHI susceptibility in the present study, regardless of the adjustments. The results of allele analyses are presented in Table 2. The genetic distributions of all SNPs in the present study followed Hardy-Weinberg equilibrium.

**Table 2:** Relationships between alleles of HSP70 genetic polymorphisms and age-related hearing impairment

Gene	SNP	Allele	ARHI	Control	Crude		Adjusted^a^	
					OR (95% CI)	p	OR (95% CI)	p
HSPA1A	rs1043618	G	202 (71.1%)	209 (73.1%)	1.000		1.000	
		C	82 (28.9%)	77 (26.9%)	1.102 (0.764–1.589)	0.604	1.111 (0.763–1.618)	0.583
HSPA1L	rs2227956	A	227 (79.4%)	241 (83.1%)	1.000		1.000	
		G	59 (20.6%)	49 (16.9%)	1.278 (0.840–1.946)	0.252	1.333 (0.863–2.508)	0.195
	rs2075800	C	176 (60.7%)	181 (62.8%)	1.000		1.000	
		T	114 (39.3%)	107 (37.2%)	1.096 (0.783–1.533)	0.594	1.033 (0.730–1.461)	0.854
HSPA1B	rs2763979	C	214 (74.8%)	171 (60.6%)	1.000		1.000	
		T	72 (25.2%)	112 (39.4%)	0.517 (0.361–0.739)	< 0.001^**^	0.520 (0.360–0.751)	< 0.001^**^

a: adjusted for sex and noise exposure

**: p < 0.001

### Genotype analysis

Multiple genetic models, including additive, dominant, and recessive inheritance patterns, were used in the genotype analysis. Similar to the allele analysis, genotypes of the SNPs rs1043618, rs2227956 and rs2075800 were not associated with ARHI, regardless of the adjustments for sex and noise exposure. The participants with the “CT” and “TT” genotypes at rs2763979 had a decreased risk of ARHI in the additive and dominant genetic model analyses. The results indicated that the “T” allele of SNP rs2763979 was associated with a decreased risk of ARHI in a dominant inheritance pattern. The results of genotype analyses are presented in Table 3.

**Table 3:** Relationships between genotypes of HSP70 genetic polymorphisms and age-related hearing impairment

Gene	SNP	Model	Genotype	ARHI	Control	Crude		Adjusted^a^	
						OR (95% CI)	p	OR (95% CI)	p
HSPA1A	rs1043618	Additive	GG	71 (50.0%)	76 (53.1%)	1.000		1.000	
			CG	60 (42.3%)	57 (39.9%)	1.127 (0.693–1.832)	0.630	1.185 (0.718–1.956)	0.506
			CC	11 (7.7%)	10 (7.0%)	1.177 (0.471–2.941)	0.727	1.116 (0.435–2.862)	0.819
		Dominant	GG	71 (50.0%)	76 (53.1%)	1.000			
			CC + CG	71 (50.0%)	67 (46.9%)	1.134 (0.713–1.806)	0.595	1.175 (0.728–1.896)	0.510
		Recessive	GG + CG	131 (93.0%)	133 (92.3%)	1.000		1.000	
			CC	11 (7.0%)	10 (7.7%)	1.117 (0.459–2.719)	0.808	1.036 (0.415–2.584)	0.940
HSPA1L	rs2227956	Additive	AA	89 (62.2%)	99 (68.3%)	1.000		1.000	
			AG	49 (43.3%)	43 (29.7%)	1.268 (0.769–2.089)	0.352	1.350 (0.805–2.264)	0.255
			GG	5 (3.5%)	3 (2.1%)	1.854 (0.431–7.981)	0.407	1.857 (0.411–8.389)	0.421
		Dominant	AA	89 (62.2%)	99 (68.3%)	1.000		1.000	
			AG + GG	54 (37.8%)	46 (31.7%)	1.306 (0.803–2.124)	0.282	1.384 (0.837–2.290)	0.205
		Recessive	AA+AG	138 (96.5%)	142 (97.9%)	1.000		1.000	
			GG	5 (3.5%)	3 (2.1%)	1.715 (0.402–7.314)	0.466	1.683 (0.377–7.526)	0.495
	rs2075800	Additive	CC	48 (33.1%)	60 (41.7%)	1.000		1.000	
			CT	80 (55.2%)	61 (42.4%)	1.639 (0.989–2.2.716)	0.055	1.572 (0.933–2.648)	0.089
			TT	17 (11.7%)	23 (16.0%)	0.924 (0.444–1.923)	0.832	0.812 (0.382–1.727)	0.588
		Dominant	CC	48 (33.1%)	60 (41.7%)	1.000		1.000	
			CT + TT	97 (66.9%)	84 (58.3%)	1.443 (0.894–2.330)	0.133	1.357 (0.828–2.224)	0.226
		Recessive	CC + CT	128 (88.3%)	121 (84.0%)	1.000		1.000	
			TT	17 (11.7%)	23 (16.0%)	0.699 (0.356–1.371)	0.297	0.627 (0.313–1.256)	0.188
HSPA1B	rs2763979	Additive	CC	82 (57.3%)	50 (35.2%)	1.000		1.000	
			**CT**	**50 (35.0%)**	**72 (50.7%)**	**0.423 (0.256–0.701)**	0.001^**^	**0.425 (0.253–0.714)**	0.001^**^
			**TT**	**11 (7.7%)**	**20 (14.1%)**	**0.335 (0.148–0.758)**	0.009^*^	**0.337 (0.146–0.779)**	0.011^*^
		Dominant	CC	82 (57.3%)	50 (35.2%)	1.000		1.000	
			**CT + TT**	**61 (42.7%)**	**92 (64.8%)**	**0.404 (0.251–0.652)**	< 0.001^**^	**0.406 (0.248–0.664)**	< 0.001^**^
		Recessive	CC + CT	132 (92.3%)	122 (85.9%)	1.000		1.000	
			TT	11 (7.7%)	20 (14.1%)	0.508 (0.234–1.104)	0.087	0.512 (0.231–1.136)	0.100

a: adjusted for sex and noise exposure

*: p < 0.05

**: p < 0.001

#### Haplotype analysis

Four common haplotypes in a block of SNPs rs2075800, rs2227956 and rs1043618 were found and analyzed (Table 4). Among the haplotypes, the “CAG” haplotype had a higher proportion in the control group than in the cases. The analysis by Haploview showed that the “CAG” haplotype was associated with ARHI susceptibility (chi-square; *p* = 0.015) and with a decreased risk for ARHI development (OR = 0.526, 95% CI = 0.320–0.865, *p* = 0.010).

**Table 4:** Haplotypes of target SNPs

Haplotypes of Block 1^a^	Population Frequency	Case/Control Frequencies	OR^b^	95% CI	*P* value
TAG	0.380	0.390/0.369	1.098	0.782–1 .540	0.589
CAC	0.281	0.287/0.275	1.064	0.735–1.540	0.741
CGG	0.186	0.207/0.165	1.346	0.872–2.080	0.178
**CAG**	**0.151**	**0.115/0.187**	**0.526**	**0.320–0.865**	0.010^*^

a: SNP block of rs2075800/rs2227956/rs1043618

b: each haplotype versus all others

*: p < 0.05

## Discussion

In the present study, we investigated the relationship between four SNPs in the HSP70 gene and ARHI in an elderly population. Our results revealed that one SNP was associated with ARHI susceptibility. The minor “T” allele of rs2763979 (HSPA1B) showed a decreased risk of ARHI (adjusted OR = 0.520, 95% CI = 0.360–0.751; *p* < 0.001). The protective effect of the “T” allele of rs2763979 from ARHI was in the dominant hereditary pattern (CT + TT versus CC; adjusted OR = 0.406, 95% CI = 0.248–0.664; p < 0.001). These results indicate that HSPA1B may be associated with human susceptibility to ARHI. In our earlier study on the association of HSPA1B SNP rs2763979 with NIHL, we found similar results in which the participants with the “T” allele of rs2763979 tended to be more resistant to NIHL. However, the association of rs2763979 with NIHL did not reach statistical significance due to the small sample size of NIHL cases in that study [12].

Some studies analyzed the associations of HSPA1B SNP rs2763979 with certain diseases other than hearing impairments. In a study in South India, subjects with the “A” allele, which is the complementary of the “T” allele in the base-paired strand of DNA of SNP rs2763979 (+ 1538 A/G), showed a relatively lower risk of albuminuria in patients with type 2 diabetes mellitus [18]. Another study on chronic obstructive pulmonary disease (COPD) found that the dominant model of the “T” allele of rs2763979 (i.e., CT + TT versus CC) increased the risk of COPD in smokers (OR = 1.60, *p* = 0.007). However, the result would be unreliable and inconclusive due to the deviation from Hardy-Weinberg equilibrium [19]. In a Korean study on alopecia areata (AA), the authors found that the promoter SNP rs2763979 had little effect on the onset of AA [20]. These studies indicated that the effects of SNP rs2763979 might be disease specific.

Although single SNP analysis of HSPA1L and HSPA1A in the present study showed no significant association with ARHI, the haplotype analysis of these SNPs (rs2075800/rs2227956/rs1043618) revealed some relationship to ARHI. Participants with the “CAG” haplotype have a decreased risk of ARHI. In our earlier studies on the associations of HSP70 genetic polymorphisms (HSPA1L/HSPA1A/HSPA1B: rs2075800/rs1043618/rs2763979) with NIHL and sudden sensorineural hearing loss (SSNHL), different haplotypes were associated with diverse risks of these hearing impairments [12, 21]. In the present study, the effect of HSPA1B rs2763979 on ARHI was so enormous that it was excluded from the haplotype analysis by Haploview software. Different haplotypes of HSPA1L/HSPA1A/HSPA1B SNP combinations were associated with variable etiologies of hearing impairment. Accordingly, we speculated that HSP70 might interact with different targets in the pathogenesis of NIHL, SSNHL, and ARHI before the mechanisms of these hearing impairments enter the common pathway of inner ear cell apoptosis/necrosis.

Several limitations exist in the present study. First, the detailed time and dosage of noise exposure could not be ascertained. More male participants had occupational noise exposure and hearing loss than females. The interaction of sex and noise exposure on ARHI might be another confounding factor that should be taken into consideration. As important confounders in ARHI, we adjusted for noise exposure history and sex in the regression analyses. Second, the sample size was relatively small in the present study. To magnify the difference in hearing between the case and control groups, we chose the best and worst quartiles of PTA for analysis. To minimize the interference of age, we used age-matched selection while grouping. Both selections may decrease the sample size for analysis. However, we believe such selections may demonstrate a more reliable result. Another limitation was that other potential risk factors for hearing loss (e.g., smoking habits, chronic medical diseases) could not be integrated and analyzed in the present study.

In summary, four SNPs that are located in three genes of the HSP70 family in an elderly population were analyzed. This investigation is the first report of a potential contribution of HSP70 genetic variants to susceptibility to ARHI. Our results support the association of genetic polymorphisms in the HSP70 gene with an individual’s susceptibility to ARHI. The HSP70 family may play an important role in the development of ARHI. Further studies to replicate our results are necessary, and further functional analyses of SNP rs2763979 in the HSP70 gene in ARHI are warranted.

## Conclusion

The results of this study support the influence of genetic polymorphisms of HSP70 on the risk of ARHI in an elderly population. Furthermore, the minor “T” allele of HSPA1B rs2763979 may be a protective allele for ARHI. However, the functional role of SNP rs2763979 in ARHI requires further investigation.

## Data Availability

Not applicable.
